# Convergence insufficiency as a predictor of poor prognosis after acute mild traumatic brain injury

**DOI:** 10.1186/s12245-024-00747-6

**Published:** 2024-11-01

**Authors:** Kavya Devani, Neera Kapoor, Latha Ganti

**Affiliations:** 1 Vista Ridge High School, Cedar Park, TX 78613 USA; 2 New York University’s Grossman School of Medicine, New York, NY 10016 USA; 3 Orlando College of Osteopathic Medicine, Winter, FL 34787 USA; 4 Warren Alpert Medical School of Brown University, Providence, RI 02903 USA

**Keywords:** Convergence insufficiency, Traumatic brain injury, Head injury, Post-concussive syndrome, Extracranial manifestations of TBI

## Abstract

**Background:**

Mild traumatic brain injury (mTBI) is becoming a more common emergency department (ED) presentation. Towards this end, many types of testing in the acute setting are being investigated. One of these is screening for convergence insufficiency (CI) symptoms. These are common problems reported by patients with mTBI, but such oculomotor testing is rarely performed in the ED.

**Objective:**

To assess the feasibility of convergence insufficiency screening in the ED and investigate whether CI is associated with adverse events such as post-concussive symptoms or hospital admission.

**Methods:**

Written informed consent was obtained from patients age 18 years or older who experienced a mild head injury from any mechanism resulting in an mTBI. Patients underwent screening for CI symptoms using a standardized instrument of 15 questions, known as the convergence insufficiency symptom survey (CISS), with responses based on the Likert scale. These data were correlated to outcomes of hospital admission, occurrence of post-concussive symptoms, and 30-day hospital re-admission.

**Results:**

A total of 116 patients were prospectively enrolled, of which 58 were male. The median age was 31 years, with a range of 18 to 95 years of age. The median CISS score was 13, with an interquartile range (IQR) of 6 to 21 and an overall range of 0 to 53. Females presented with a median CISS score of 14, which was higher compared to the male median score of 10. The higher the CISS score, the more likely the patient was to be admitted to the hospital (*p *= 0.0378), develop symptoms of post-concussive syndrome at 30-day follow up (*p *= 0.0322), and be readmitted within 30 days (*p *= 0.0098).

**Conclusions:**

Screening for CI symptoms using the CISS can be a solid adjunct in the evaluation of mTBI in the ED. The CISS is easy and fast to administer, and it is a useful tool to stratify patients in terms of who is at the highest risk of developing complications related to the mTBI.

## Introduction

In 2020, the CDC documented 214,110 hospitalizations related to traumatic brain injury (TBI), with an additional 69,473 TBI-related deaths reported in 2021 [1]. This breaks down to a daily average of 586 hospitalizations and 10 deaths attributed to TBI, underscoring the widespread and serious nature of this injury [1]. Traumatic brain injury can be categorized into non-penetrating and penetrating forms. Non-penetrating TBI occurs when the brain sustains damage from a blow, bump, or jolt to the head or body. Conversely, penetrating TBI involves physical harm to the brain by passing through the skull [2]. While individuals of all ages, races, and genders can experience TBI, certain groups face a higher risk of enduring long-lasting effects due to previous trauma or limited access to advanced healthcare, as reported by the CDC [1]. Falls, firearm-related incidents, motor vehicle accidents, and assaults are common causes of TBI, with falls alone accounting for nearly half of TBI-related hospitalizations [3]. Approximately three quarters of TBIs that occur each year are concussions or other forms of mild TBI [4].

TBI can manifest through physical, cognitive, and perceptual/sensory symptoms [3]. Physical symptoms encompass headaches, vision changes, seizures, fluids from the ears or nose, nausea, or any neurological manifestation [3, 4]. Cognitive symptoms include alterations in consciousness levels, confusion, changes in sleep patterns, or behavioral shifts [3]. Changes in perception and sensations refer to alterations in the ability to interpret and process senses such as balance, taste, vision, hearing, and emotion [3, 4]. These symptoms arise from factors such as swelling, tearing, bruising, bleeding, or any alterations in the brain. The primary effects of TBI include diffuse axonal injury, concussion, hematomas, and contusions, as well as skull fractures, chronic traumatic encephalopathy, or post-traumatic dementia [3].

TBI severity is typically categorized as mild, moderate and severe based on the Glasgow Coma Scale (GCS), which assigns a score ranging from 3 to 15. This scale evaluates eye response (score out of 4), verbal response (score out of 5), and motor response (score out of 6) [5]. A higher score indicates a less severe injury. Specifically, a GCS score of 3 to 8 denotes severe TBI, 9 to 12 indicates moderate TBI and 13 to 15 indicates mild TBI (mTBI) [5]. However, a recent study of over 2200 hundred patients who presented to the ED with a mTBI demonstrated that there is in fact, nothing “mild” about a mTBI, even with a GCS of 15, much less one that is 13 or 14 [6].

This current study focuses on mTBIs and how screening for convergence insufficiency (CI) symptoms may be an efficient tool to risk-stratify patients in an emergency department (ED) setting. CI affects the ability of both eyes to work together to form a single image, resulting from damage or dysfunction of nerves that control the eye muscles [7]. Although usually a problem that manifests in childhood, CI can occur following a brain injury as well [8]. Common symptoms include visual fatigue, double vision, headaches, or trouble concentrating [9].

This study focuses on the connection between the prognosis as well as symptom development regarding mTBI and the presentation of CI symptoms at the acute stage of mTBI at the ED. Since CI is a common sequela of mTBI, the goal of this study is to understand if CI symptomatology could be used as a predictor of prognosis to risk-stratify mTBI patients presenting to the ED.

## Methods

This is a prospective observational study of consecutive adult patients who presented to the ED of a level I trauma facility with a mild TBI defined as a Glasgow Coma Scale (GCS) of 13–15. The mTBI must have occurred within the prior 24 h. Written informed consent was obtained from the patients to perform oculomotor testing including screening for CI symptoms using the CISS, in addition to their routine ED care.

**Fig. 1:**
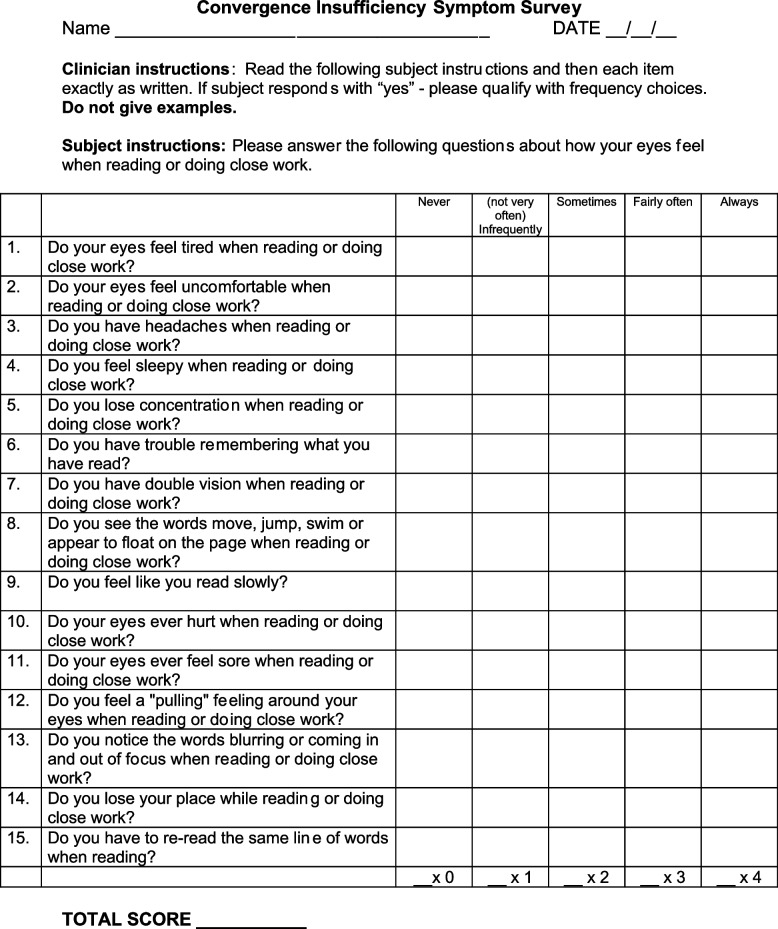
Validated Convergence Insufficiency Symptom Survey [10]

The CISS is a validated 15-question instrument based on a Likert scale to assess the likelihood of CI, with “never” being 0 points and “always” being 4 points Fig. 1 [10]. Greater symptomatology related to CI is associated with a CISS score of 21 points or higher for adults and 16 and higher for children [10].

Consent also included follow-up telephone visits at 7 (3- to 15-day interval, defined as “early follow-up”) and 30 days (30- to 45-day interval, defined as “late follow up”). Telephone follow-up visits included scripted questionnaires to assess whether patients had any symptoms suggestive of post-concussion syndrome (PCS), including headache, vomiting, dizziness, tinnitus, sensitivity to light, sensitivity to noise, numbness or tingling, blurred vision, diplopia, flashing lights, drowsiness, fatigue or lethargy, sadness or depression, nervousness or irritability, difficulty concentrating or remembering, sleeping problems, as well as feeling “slowed down,” “in a fog” or “dazed.” An affirmative response to any of these questions was considered to indicate the presence of PCS [11].

Data were entered into REDCap, a secure data collection tool that meets HIPAA compliance standards [12]. Statistical analyses were performed in JMP 16.0 for the Mac (Cary, NC) [13]. This study was conducted within the TBI ADAPTER trial [14] and was approved by the medical school’s Institutional Review Board. Results of concurrent neurocognitive testing performed have been previously reported [15].

## Results

**Fig. 2:**
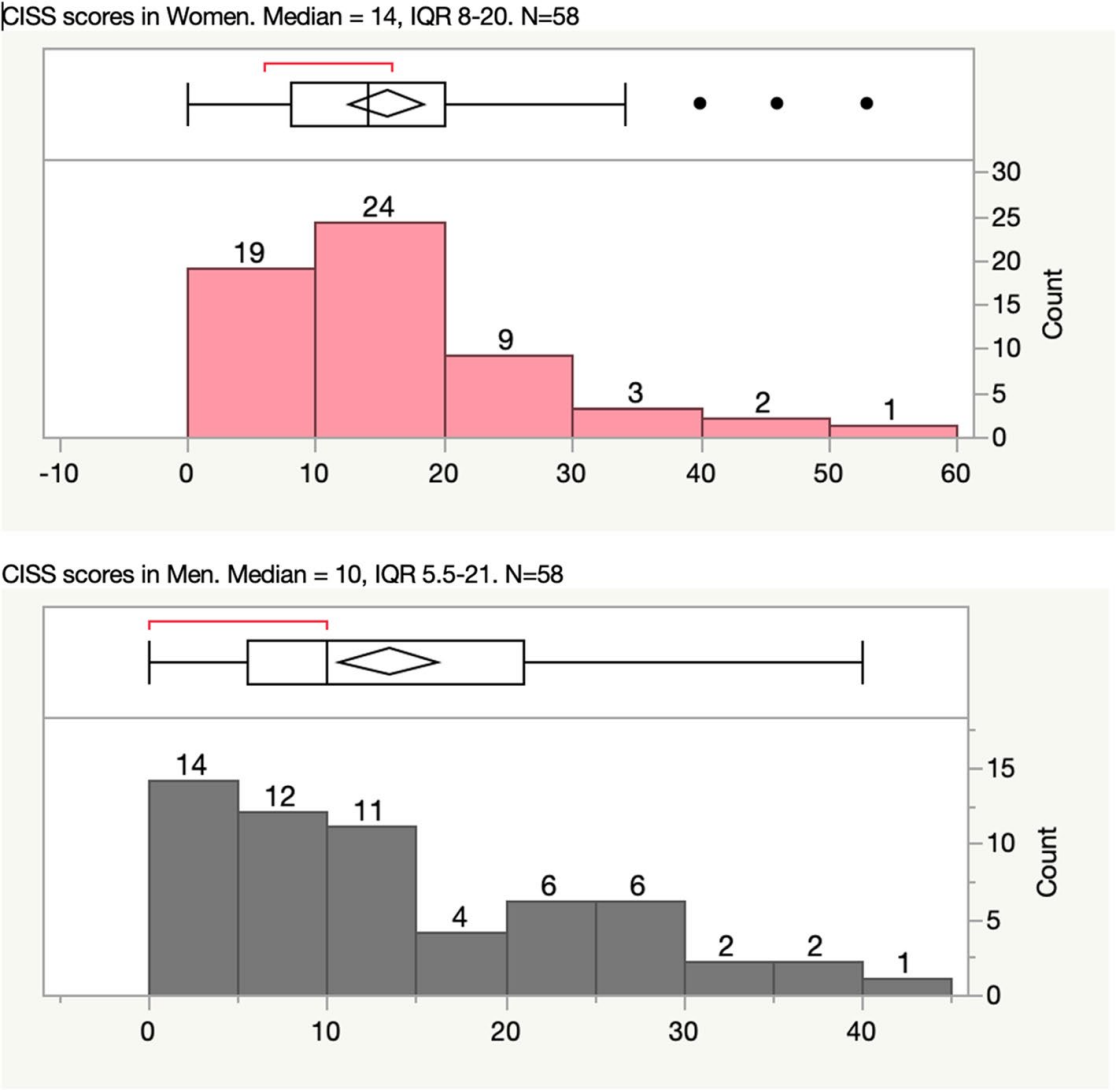
CISS score distribution amongst men and women

The cohort consisted of 116 patients with mTBI, age range 18–95 years, with a median of 30.5 years, and IQR of 21 to 50. The data were evenly split, with 58 males and 58 females. The racial distribution was 75% White, 19% Black, 3% Hispanic, and 3% other. The IQR for the score distribution was 6–21, with the majority of patients CISS score less than 21. Females showed a higher median CISS score of 14 while males had a median CISS score of 10 (Fig. 2). A total of 32 patients (28%) had a CISS score *≥* 21.

**Fig. 3:**
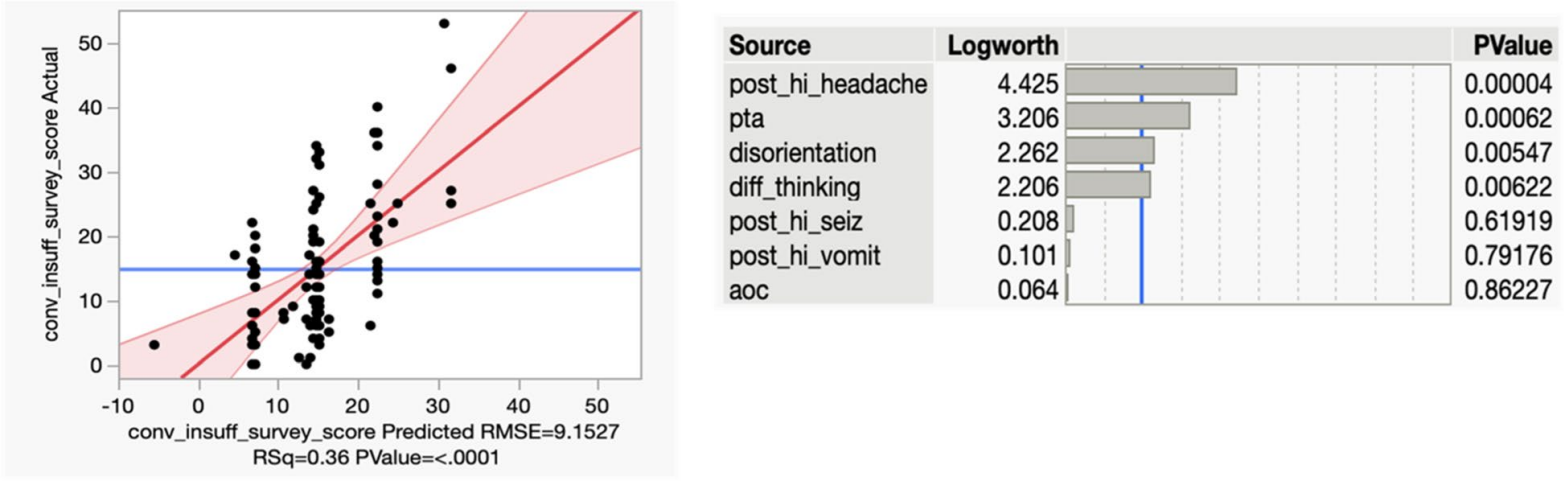
Multivariate model depicting the correlation of CISS score to Post Concussive Syndrome after mild TBI

Figure 3 depicts a multivariate model that demonstrates a higher CISS score correlates with having post-concussive syndrome (PCS). Specifically, symptoms of headache (*P* = 0.00004), post-traumatic amnesia (*P* = 0.00062), disorientation (*P* = 0.00547), and difference in thinking or “fog” (*P* = 0.00622). A higher CISS score was not significantly correlated with post-mTBI vomiting or altered state of consciousness. This model was robust, with an overall p-value of < 0.0001 and a coefficient of determination (R^2^) value of 36%.

**Fig. 4:**
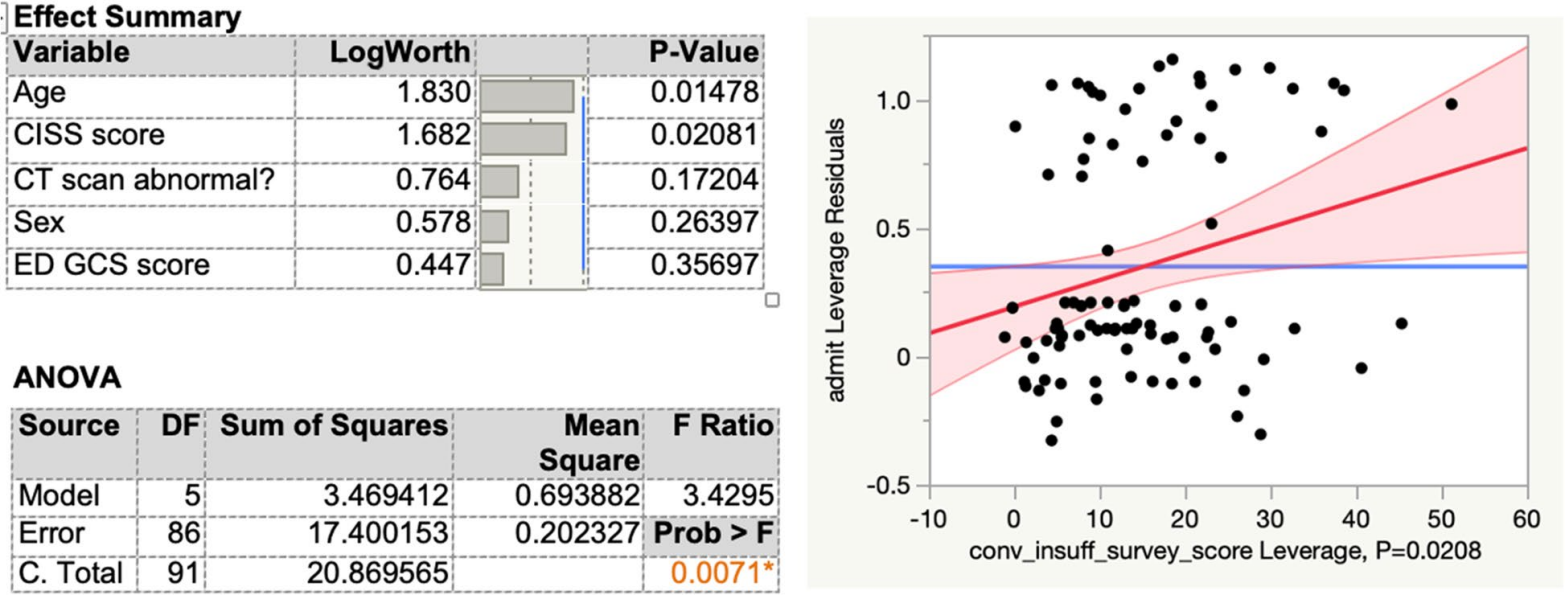
Multivariate model depicting the correlation of CISS score to hospital admission after mild TBI

A second multivariate model was built to analyze a potential correlation between the CISS score and the likelihood of being admitted for persisting symptoms related to mTBI Fig. 4. The model included age, sex, whether the initial computed tomography (CT) scan was abnormal, the ED’s GCS score and the ED’s CISS score. Figure 3 demonstrates that CISS is significantly correlated to hospital admission in this model, with a p-value of 0.02081. This model yielded an overall p-value of 0.0071, and a coefficient of determination (R^2^) value of 17%.

**Fig. 5:**
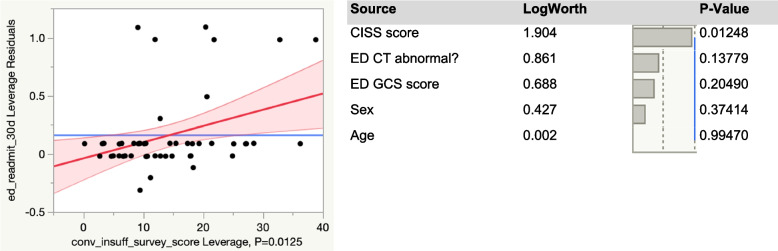
Multivariate model depicting the correlation of CISS score to hospital re-admission within 30 days after mild TBI

A third multivariate model was built to analyze a potential correlation between the CISS score and the likelihood of being re-admitted within 30 days for persisting symptoms related to the mTBI Fig. 5. The model included age, sex, whether the initial computed tomography (CT) scan was abnormal, the ED’s GCS score and the ED’s CISS score. Figure 4 demonstrates that CISS is significantly correlated to hospital admission in this model, with a p-value of 0.01248. This model was robust, with an overall p-value of 0.0118, and a coefficient of determination (R^2^) value of 28%.

## Discussion

This study aimed to assess the viability of administering the CISS in the ED and determine whether individuals over 18 years of age with a CISS greater than 21 were more prone to experiencing post-concussive symptoms and/or being admitted or re-admitted within 30 days. The results revealed a positive correlation between the CISS score and the development of post-concussive symptoms at a 30- to 45-day follow-up, as well as being admitted to the hospital at the initial ED visit and within 30 days thereafter. These data suggest that screening for CI symptoms with the CISS could be an effective tool for risk-stratifying patients, especially given that the presentation of mTBI has become more prevalent in the ED setting.

A prior study of 72 TBI patients who were followed up at three years post-injury revealed a presence of vergence dysfunction in approximately 42% of these patients. In addition, these investigators reported that CI was associated with cognitive disturbances (*p* < 0.005), longer periods of coma (*p* < 0.001), and an inability of patients to find work in the open market (*p* < 0.01) [16]. Another study of 160 TBI patients reported that 90% presented with sensorimotor vision deficits, with accommodative and vergence deficits being the most common [17]. This suggests that CI portends a poor prognosis or further complications after a TBI. A review of visual impairments in the first year after traumatic brain injury encompassing 18 studies found that visual impairment negatively impacts independence in mobility and activities of daily living [18]. The investigators noted that the most common visual impairments seen include blurred vision, reading problems, diplopia, eyestrain, dizziness or disequilibrium in visually crowded environments, visual field defects, light sensitivity, and color blindness [19].

The significant association found between convergence insufficiency, hospital admission rates, and persisting post-concussive symptoms is biologically reasonable given the anatomy and physiology of extraocular motility. The nuclei for the three cranial nerves (oculomotor, trochlear, and abducens) innervating extraocular motility originate in the brain stem at the level of the midbrain. In addition to the midbrain and these three brain stem nuclei, other neurological areas that affect vergence include the frontal eye fields, mesencephalic reticular formation, medial longitudinal fasciculus, and cerebellum. Given these neuro-anatomical connections for vergence extraocular motility, it seems probable that mTBI might impact one or more of these areas impairing extraocular motility and possibly resulting in CI. Disruption to other neural tissues, vasculature, and cellular structures from an mTBI, beyond the discrete injury itself, lends credence to the fact that having a first concussion increases the probability of subsequent ones [20].

As TBI cases presenting to the ED continue to rise in numbers, it is of the utmost importance to identify factors that could help determine the need for early intervention. If supported by further analysis, screening for CI symptoms could guide triage for higher-level care and immediate intervention. It could serve as an identification tool for those at risk of post-concussive symptoms and other complications, offering care pathways to optimize both patient outcomes and healthcare utilization. Substantive work has been performed regarding vision rehabilitation of vision deficits following mTBI [21–30], but larger-scale randomized controlled trials are indicated.

## Conclusion

The current study demonstrates the feasibility of screening for CI symptoms in a busy ED setting for patients who experience mTBI or concussion. A higher CISS demonstrated a positive correlation with the development of PCS, being admitted to the hospital, and being at risk for re-admission within 30 days. Though more research is needed, this work offers a foundational idea to be built upon to target interventions toward at-risk mTBI patients in fast-paced, high-volume environments, such as the Emergency Department.

## Data Availability

No datasets were generated or analysed during the current study.
